# Alteration of Neural Stem Cell Functions in Ataxia and Male Sterility Mice: A Possible Role of β-Tubulin Glutamylation in Neurodegeneration

**DOI:** 10.3390/cells10010155

**Published:** 2021-01-14

**Authors:** Abdullah Md. Sheikh, Shozo Yano, Shatera Tabassum, Koji Omura, Asuka Araki, Shingo Mitaki, Yoshie Ito, Shuai Huang, Atsushi Nagai

**Affiliations:** 1 Department of Laboratory Medicine, Shimane University Faculty of Medicine, 89-1 Enya Cho, Izumo 693-8501, Japan; syano@med.shimane-u.ac.jp (S.Y.); tabassum@med.shimane-u.ac.jp (S.T.); 2 Department of Organ Pathology, Shimane University Faculty of Medicine, 89-1 Enya Cho, Izumo 693-8501, Japan; kou00@med.shimane-u.ac.jp (K.O.); asuka@med.shimane-u.ac.jp (A.A.); 3 Department of Neurology, Shimane University Faculty of Medicine, 89-1 Enya Cho, Izumo 693-8501, Japan; shingomi@med.shimane-u.ac.jp (S.M.); yo.ito@med.shimane-u.ac.jp (Y.I.); anagai@med.shimane-u.ac.jp (A.N.); 4 Department of Orthopedic Surgery, The Second Affiliated Hospital of Guangzhou Medical University, 250 Changgangdong Road, Guangzhou 510260, China; huang-shuai@hotmail.com

**Keywords:** AMS mouse, NSC, deglutamylation, β-tubulin, MAP2, NNA1

## Abstract

Ataxia and Male Sterility (AMS) is a mutant mouse strain that contains a missense mutation in the coding region of *Nna1*, a gene that encodes a deglutamylase. AMS mice exhibit early cerebellar Purkinje cell degeneration and an ataxic phenotype in an autosomal recessive manner. To understand the underlying mechanism, we generated neuronal stem cell (NSC) lines from wild-type (NMW7), *Nna1* mutation heterozygous (NME), and *Nna1* mutation homozygous (NMO1) mouse brains. The NNA1 levels were decreased, and the glutamylated tubulin levels were increased in NMO1 cultures as well as in the cerebellum of AMS mice at both 15 and 30 days of age. However, total β-tubulin protein levels were not altered in the AMS cerebellum. In NMO1 neurosphere cultures, β-tubulin protein levels were increased without changes at the transcriptional level. NMO1 grew faster than other NSC lines, and some of the neurospheres were attached to the plate after 3 days. Immunostaining revealed that SOX2 and nestin levels were decreased in NMO1 neurospheres and that the neuronal differentiation potentials were reduced in NMO1 cells compared to NME or NMW7 cells. These results demonstrate that the AMS mutation decreased the NNA1 levels and increased glutamylation in the cerebellum of AMS mice. The observed changes in glutamylation might alter NSC properties and the neuron maturation process, leading to Purkinje cell death in AMS mice.

## 1. Introduction

The ataxia and male sterility (AMS) mouse is a mutant strain that spontaneously arose from autoimmune-prone MRL/*lpr* mouse [1]. The putative disease-causing mutation is inherited in an autosomal recessive manner. Since the disease phenotype was existed in this mouse strain even in the absence of *lpr* mutation, the disease phenotype was demonstrated to be independent of this mutation [2,3]. Ataxia is a major clinical feature of this mouse strain that starts to appear at approximately 3 weeks of age [1]. Initially, mice show a subtle sway of the trunk and failure to maintain a still posture. Subsequently, a mild-to-moderate ataxic gait appears, which is maintained throughout life. Later, a missense mutation in the 17th exon of the *nervous system nuclear protein induced by axotomy protein 1* (*Nna1*) gene was revealed, causing a substitution of amino acid 808 from arginine to proline [4]. Another name for the *Nna1* gene is *ATP/GTP binding protein 1* (*Agtpbp-1*). Previous studies reported that mutations in the *Nna1* gene can induce ataxia in mice starting from an early age [5,6,7]. The main pathological feature of these mouse strains is the early degeneration of cerebellar Purkinje cells (PC); hence, these mice are called PC degeneration (PCD) mice. AMS mice also show early PC degeneration, which explains the clinical feature of ataxia [1]. At 4.5 months of age, about a 25% decrease in total brain weight can be observed in AMS mice, and their cerebellum is smaller compared to wild-type littermates [1]. A total loss of PC somata and dendrites can reduce the volume of the molecular layers. Subsequent examination demonstrated the degeneration and shrinkage of the granular layer as well [3]. Hence, the combined degeneration of PCs and granule cells in this mouse strain leads to a reduced cerebellum and altered cerebellar control over motor functions.

Since *Nna1* is the only mutated gene in AMS mice and NNA1 expression is relatively high in the cerebellum [8], alteration of the protein function could play a vital role in the degeneration process of cerebellar neurons [9]. In previous studies, it was demonstrated that the NNA1 protein is localized in mitochondria, where it is essentially involved in bioenergetics. The loss-of-function of NNA1 causes mitochondrial dysfunction and induces a PCD phenotype [10]. Indeed, hippocampal neurons of AMS mice showed increased vulnerability during hypoxia due to oxidative stress, suggesting that mitochondrial function might be affected here due to *Nna1* mutation [11]. However, hippocampal neurons do not show signs of early degeneration like cerebellar PC. These findings suggest functional differences between these neuron types and distinct roles for NNA1 in their functions. NNA1 also shows carboxypeptidase activity [8]. Importantly, NNA1 catalyzes the deglutamylation of polyglutamylated proteins [12]. Polyglutamylation is a posttranslational protein modification where strings of polyglutamate are added onto the gamma carboxyl groups of any of several glutamine residues near the C-terminus of either α- or β-tubulin [13]. Increased tubulin polyglutamylation was observed in PCD mice strains [14], suggesting that this NNA1 function could be indispensable for the pathology. Indeed, NNA1 was found to regulate mitochondrial motility through deglutamylation of tubulin [14]. Motility is important for mitochondrial fusion, and the loss of NNA1-mediated mitochondrial fusion accounts for the exquisite vulnerability of Purkinje neurons in PCD mice [14]. However, if the alteration of mitochondrial functions was the sole cause of PC death, then neurons of other areas like the hippocampus, where NNA1 expression is high, should be affected similarly. Yet, hippocampal neurons do not show similar patterns of neurodegeneration like PCs in AMS mice despite a high expression of NNA1 [8,11]. This suggests the existence of additional mechanisms that are important for PC neurodegeneration in AMS mice.

Modulation of the cytoskeleton is involved in various cellular processes including cell division, homing, and polarity during the developmental stages of neurons [15]. As a component of cytoskeleton, microtubules have the ability to regulate these cellular processes. The main components of microtubules are α- and β-tubulin [16]. In microtubules, extensive posttranslational modifications of tubulins, such as phosphorylation, glutamylation, glycylation, tyrosinylation, and acetylation are required [17,18]. Such posttranslational modifications determine the characteristics of microtubules that perform different types of functions depending on the cellular processes. Hence, posttranslational modifications of tubulins are tightly controlled to regulate its function and dynamics required for particular cellular processes. In postmitotic neurons, microtubule dynamics play an important role in development and maturation. For example, during the migration of newly formed neurons, microtubules were found to be indispensable [19]. Besides, during maturation, microtubules play an important role in neurite outgrowth [20,21]. Functioning as a deglutamylase, NNA1 could influence such microtubule dynamics and maturation of neurons, and alteration of its function might affect neurodevelopmental processes. A recent study showed that mutations in the *NNA1* gene are indeed linked to human infantile-onset neurodegeneration, where dysregulation of tubulin polyglutamylation is evident [22]. Another report demonstrated that the lack of NNA1 affects microtubule dynamics and flexibility, defects that contribute to morphological alterations of PCs [23] and lead to progressive cerebellar degeneration. Since PC degeneration occurs during early postnatal development of the cerebellum and microtubule dynamics are important for the postmitotic development of cerebellar neurons, we sought to investigate the role of NNA1 during the early development of neurons in AMS mice. One way to examine the influence of NNA1 on neuronal development and maturation is to use cultured neural stem cells (NSCs) and to evaluate the role of the protein in these processes.

Thus, to investigate the role of NNA1 on neuronal development and maturation, we generated NSC lines from AMS mice carrying homozygous *Nna1* mutation, heterozygous mice, and their wild-type littermates and checked their characteristics and differentiation potential. We found that the characteristics and maturation potential of the AMS NSC line were altered along with increased β-tubulin levels and tubulin glutamylation. Such altered neuronal maturation could be important for neuronal degeneration in AMS mice.

## 2. Materials and Methods

### 2.1. Materials

Dulbecco’s modified Eagle medium (DMEM) and Hank’s Balanced Salt Solution were purchased from Wako Pure Chemicals (Richmond, VA, USA), and fetal bovine serum (FBS) was from Gibco (Invitrogen, Carlsbad, CA, USA). F12 ham was purchased from Sigma-Aldrich (St. Lois, MO, USA). Epidermal growth factor (EGF) and basic fibroblast growth factor (bFGF) were from PeproTech (Rocky Hill, NJ, USA). Cell culture plates and dishes were from Falcon (Corning, Oneonta, NY, USA). Permanox chamber slides, N2 supplement, and B27 supplement were purchased from Thermo Fisher (Waltham, MA, USA). Pre-stained protein size marker for Western blotting was purchased from NIPPON Genetics EUROPE GmbH (Duren, Germany), and 4–20% Tris–glycine polyacrylamide gel was from Bio-Rad (Hercules, CA, USA). Polyvinylidene difluoride (PVDF) membrane was obtained from Millipore (Billerica, MA, USA).

### 2.2. Genotyping

The genotyping of AMS mice was performed as described previously [11]. In brief, DNA was isolated from the tail, the spleen, or cultured cells using DNeasy blood and tissue isolation kit (Qiagen, Hilden, Germany) following the manufacturer’s instruction. Then, a DNA fragment of the *Nna1* gene was PCR amplified, and the genotype was determined by restriction enzyme digestion using BstB1 (New England BioLab, Ipswich, MA, USA) that digested non-mutated DNA.

### 2.3. Cell Culture

#### Isolation and Generation of Embryonic NSC Lines

All experimental protocols and procedures were approved by the ethics committee of the School of Medicine at Shimane University. All animal procedures were done according to the guidelines of the Animal Institute, Shimane University. Heterozygous mice carrying the *Nna1* mutation were mated. At 14.5 days of gestation, a pregnant mouse was deeply anesthetized, and the fetuses were collected under aseptic conditions. Fetal brain cortical tissue was isolated under a dissection microscope in a cell culture bench. The spleen was collected to isolate DNA for genotyping. The tissue was cut into small pieces in DMEM using a scalpel. After removing DMEM by centrifugation, the tissue was re-suspended in 0.25% trypsin and DNAse1 and was incubated in a water bath at 37 °C for 10 min. To neutralize trypsin, FBS was added, and the suspension was centrifuged to collect the cells. After washing with DMEM, the cells were plated in a tissue culture dish in complete media (high glucose DMEM: F12 ham 1:1, 2% FBS, N2 supplement, bFGF 20 ng/mL, and EGF 20 ng/mL).

When the cultured cells showed features of NSCs, the cells were infected with a lentiviral vector expressing small and large T antigen (pLenti CMV/TO SV40 small + Large T). pLenti CMV/TO SV40 small + Large T (w612-1) was a gift from Dr. Eric Campeau (Addgene plasmid # 22298; http://n2t.net/addgene:22298; RRID:Addgene_22298). Immortalization of NSCs was carried out according to the guidelines approved by the ethics committee of the School of Medicine at Shimane University. After about 10 subcultures, cells were plated into 100-mm cell culture dishes at low concentrations to generate colonies from single cells. Several colonies were picked up and expanded to get a homogenous culture. The cells were genotyped for *Nna1*, and *small and large T antigens*. Then, neurosphere assay was done for all colonies generated from a single fetus, and one colony among them that showed best neurosphere formation properties was selected, expanded, and further characterized.

### 2.4. Preparation of Neurosphere

To prepare the neurosphere, the cell lines were cultured in a non-tissue culture dish. The following medium was used to generate neurosphere, high glucose DMEM: F12 ham 1:1, bFGF 20 ng/mL, EGF 20 ng/mL, N2 supplement, and B27 supplement. For an experiment, neurospheres from all 3 NSC lines were grown simultaneously and then processed on the same day. Time-dependent changes in neurosphere morphologies were checked using a cell culture microscope. For immunostaining, neurospheres were fixed with 4% paraformaldehyde. Then, Tissue-Tek^®^ O.T.C compound (Sakura Finetek, Tokyo, Japan) was added to the neurospheres, and frozen blocks were prepared. These blocks were sectioned using a microtome.

### 2.5. Differentiation of NSCs to Neurons

For neuronal differentiation, poly-L-lysine (PL)-coated dishes were used. After preparation, the neurospheres in neurosphere culture medium were directly transferred into PL-coated dishes, and the same amount of differentiation medium (DMEM: F12 ham 1:1, B27) was added. After 6 h, when the neurospheres were attached to the bottom of the dish, the medium was removed, fresh differentiation medium was added, and the cells were cultured for another 14 days with medium changes every 2 days. For the immunocytochemistry of mature neurons, cells were differentiated on PL-coated Permanox chamber slides.

### 2.6. Immunostaining

For immunostaining of the cerebellum, mice were deeply anesthetized with Isofluran, and the cerebellum was isolated, fixed in formalin, and embedded in paraffin. Then, 10-μm tissue slices were prepared using a microtome. After deparaffinization, antigen retrieval was performed by boiling the tissue section in 10 mM citrate buffer (pH 6.0). After quenching the endogenous peroxidase activity, the sections were incubated in a blocking solution containing 5% normal goat or horse serum and 0.1% Triton X-100 in phosphate-buffered saline (PBS). The sections were incubated with anti-NNA1 IgG (goat, 1:200, Santa Cruz Biotechnology, Santa Cruz, CA, USA), anti-β-tubulin IgG (rabbit, 1:1000, Abcam, Cambridge, UK), anti-glutamylated tubulin IgG (mouse, 1:1000, Adipogen, Liestal, Switzerland), or anti-microtubule-associated protein 2 (MAP2) IgG (rabbit, 1:1000, Abcam), followed by incubation with the appropriate secondary antibodies. In the cases of β-tubulin, glutamylated tubulin, or MAP2, the secondary antibodies used were conjugated with fluorescein isothiocyanate (FITC) or Texas Red (Santa Cruz). Immunofluorescence staining was done after protecting the tissues from light to minimize photobleaching. In the case of NNA1, the secondary antibody was biotin-conjugated (1:100, Vector, Ingold Road, CA, USA). Then, the section was incubated with an avidin–biotin–peroxidase complex (ABC, Vector) and the immune reaction products were visualized with 3, 30-diaminobenzidine (DAB, Sigma). Stained sections were examined under a fluorescent microscope (ECLIPSE E600, NIKON, Tokyo, Japan) in a blinded manner.

For immunostaining of neurospheres, 10-μm slices were prepared from the block and antigen retrieval was performed by boiling the slices in 10 mM citrate buffer (pH 6.0). The slices were incubated in a blocking solution containing 5% normal goat or horse serum and 0.1% Triton X-100 in PBS. The sections were incubated with anti-nestin IgG (rabbit, 1:200, Abcam), anti-β-tubulin IgG (rabbit, 1:1000, Abcam), anti-glial fibrillary acidic protein (GFAP, rabbit, 1:1000, Abcam), anti-O4 IgG (rabbit, 1:1000, Cell Signaling, Beverly, MA, USA), or anti-SOX2 IgG (rabbit, 1:1000, Cell Signaling). Then, the sections were incubated with appropriate secondary antibodies conjugated with FITC or Texas Red. Stained sections were examined under a fluorescent microscope (ECLIPSE E600, NIKON) in a blinded manner.

Fluorescent intensities of the stained photomicrographs were quantified using ImageJ (NIH, Bethesda, MD, USA). After opening the photomicrograph with ImageJ, the immunoreactive fluorescent area of interest was selected and the fluorescence intensity was measured. Then, fluorescence intensities of at least five background areas were measured, averaged, and subtracted from the fluorescence intensities of the area of interest. In an experiment, at least six photomicrographs per condition were processed, and background-normalized fluorescence intensities were averaged.

### 2.7. Total RNA Isolation, Reverse Transcription, and Real-Time PCR

Total RNA was isolated from attached NSC cultures after neuronal differentiation and from neurospheres using Trizol reagent (Invitrogen) following the manufacturer’s instructions. To prepare first-strand cDNA, 2 μg of total RNA was reverse transcribed with oligo dT primers and reverse transcriptase enzyme (RiverTraAce, Toyobo, Osaka, Japan) in a 20-μL reaction mixture. To analyze the mRNA level of *β-tubulin*, real-time PCR was performed with the SyBr green PCR system (Applied Biosystem, Warrington, UK) and gene specific primers, using an ABI Prism 7900 Sequence Detector system (Applied Biosystems). The mRNA level was normalized to the corresponding Glyceraldehyde 3-phosphate dehydrogenase (GAPDH) mRNA and quantified by a relative quantification method, where a sample of an attached neuronal stem cell line from wild-type (NMW7) culture was used as the calibrator.

### 2.8. Western Blot Analysis

Western blotting was done as described previously [24]. Briefly, total protein was isolated from cultured cells or murine brain tissue using ice cold RIPA buffer (PBS, pH 7.4, 1% Nonidet p-40, 0.5% sodium deoxycholate, 0.1% SDS, 10 mg/mL PMSF, and 1 mg/mL aprotinin). To homogenize brain tissue, 20× wt/vol of RIPA buffer was used. Total protein (20–60 μg) was separated by 4–20% SDS polyacrylamide gel electrophoresis and transferred to a PVDF membrane. Then, the membrane was incubated with anti-NNA1 IgG, anti-β-tubulin IgG, anti-glutamylated tubulin IgG, anti-MAP2 IgG, or anti-nestin IgG. NNA1, β-tubulin, MAP2, and nestin were detected using the appropriate infrared-fluorophore-conjugated secondary antibodies (Li-COR, Lincoln, NE, USA). Anti-glutamylated tubulin IgG was conjugated with biotin; hence, infrared-fluorophore-conjugated streptavidin was used for detection. Immunoblotting was done after protecting the membrane from light to minimize photobleaching. Immunoreactive proteins in the membrane were detected using an infrared scanner (Li-COR). After scanning, the bands of Immunoreactive proteins were selected and densitometrically analyzed using Odyssey V3.0 (Li-COR), or ImageJ. The signal intensities of background areas of the same size were subtracted from the signals of immunoreactive proteins. β-actin was used as a loading control, and its signal intensity was used to normalize the signals of target proteins. Necessary processing of the photographs of the blots was done using ImageJ (NIH).

### 2.9. Statistical Analysis

Numerical data are presented as mean ± standard deviation (SD). Statistical differences among groups were assessed using one-way ANOVA followed by Scheffe’s post hoc test or the paired *t*-test. A *p* value < 0.05 was considered as statistically significant.

## 3. Results

### 3.1. Purkinje Cell Degeneration and NNA1 Expression in the Cerebellum of AMS Mice

**Figure 1:**
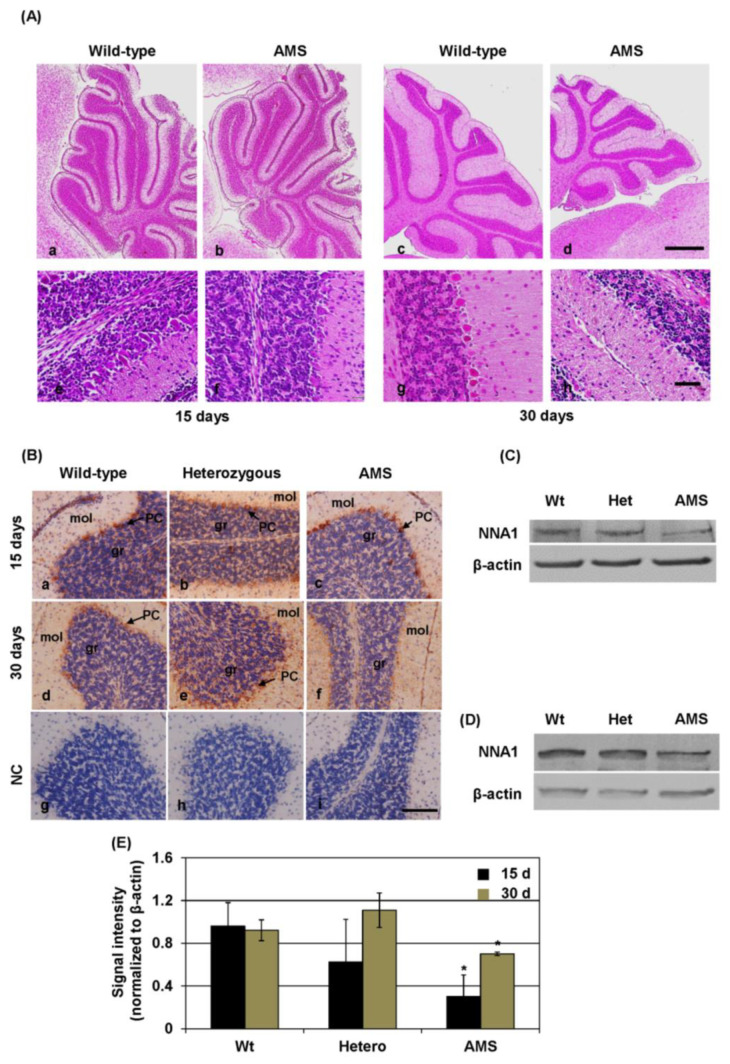
Pathological changes and NNA1 protein expression in the cerebellum of Ataxia and Male Sterility (AMS) mice: Pathological changes in the murine cerebellum were checked by hematoxylin and eosin staining. Photomicrographs of the cerebellum of wild-type (**a**,**c**,**e,g**) and AMS (**b**,**d**,**f,h**) mice at the ages of 15 (**a**,**b**,**e**,**f**) and 30 (**c**,**d**,**g,h**) days are shown in (**A**). The upper panels present photomicrographs at low magnification (×40), and the photomicrographs of the lower panels show the cerebellum at high magnification (×400). Scale bar: upper panel, 500 µm; lower panel, 50 µm. (**B**–**E**) The NNA1 protein expressions in the cerebellum of wild-type, *Nna1* mutation heterozygous, and AMS mice were evaluated using immunostaining and Western blotting. (**B**) NNA1 immunostaining is shown here, where the photomicrographs (×200 magnification) of the upper and middle panels display cerebellar samples of 15- and 30-day-old mice, respectively. Cerebellar samples of 30-day-old mice were also stained with normal goat IgG as negative controls (NC) and are shown in the lower panels. Purkinje cells (PCs) are indicated using arrows. mol = molecular layer, gr = granular layer. Scale bar, 100 μm. (**C**,**D**) The representative NNA1 Western blotting results of wild-type (Wt), *Nna1* mutation heterozygous (Het), and AMS mice at the ages of 15 (**C**) and 30 (**D**) days are shown here. β-actin was used as a loading control. (**E**) Average densitometric data of the Western blotting normalized to β-actin (*n* = 5) are presented in (**E**). Statistical significance is denoted as follows: * *p* < 0.05 vs. wild-type.

Previously, we demonstrated cerebellar PC degeneration in AMS mice [1]. In the present study, we checked the pathological changes in the cerebellum in a time-dependent manner. Hematoxylin and eosin staining showed that the size of the cerebellum was considerably smaller in AMS mice than in their wild-type littermates at 30 days of age. At 15 days, the size was similar in AMS and wild-type mice (Figure 1A). PCs were detected in the cerebellum of 15-day-old AMS mice but disappeared almost completely at 30 days. Moreover, at 6 weeks of age, the average weight of the cerebellum decreased in AMS mice to about 70% of that in wild-type littermates (Wt: 118.5 ± 3.5 mg, AMS: 82 ± 5 mg; *n* = 3 in each group; *p* < 0.001), whereas the mean weight of the cerebrum was similar (Wt: 382.9 ± 16.7 mg, AMS: 385.3 ± 5.9 mg; *n* = 3 in each group; *p* = 0.39). Immunostaining results showed that NNA1 was expressed in PCs as well as cells of the granular (identified by densely packed hematoxylin-stained nuclei) and molecular layers of the cerebellum in wild-type, *Nna1* mutation heterozygous, and AMS mice at 15 days. Since PCs were absent at 30 days in AMS mice, NNA1 was only detectable in cells of the molecular and granular layers (Figure 1B). Western blotting results showed that compared to wild-type mice, the NNA1 protein levels were decreased in the cerebellum of AMS mice at both 15 and 30 days (Figure 1C–E).

### 3.2. Establishment of Neural Stem Cell Lines from Embryonic Mouse Brains

**Figure 2:**
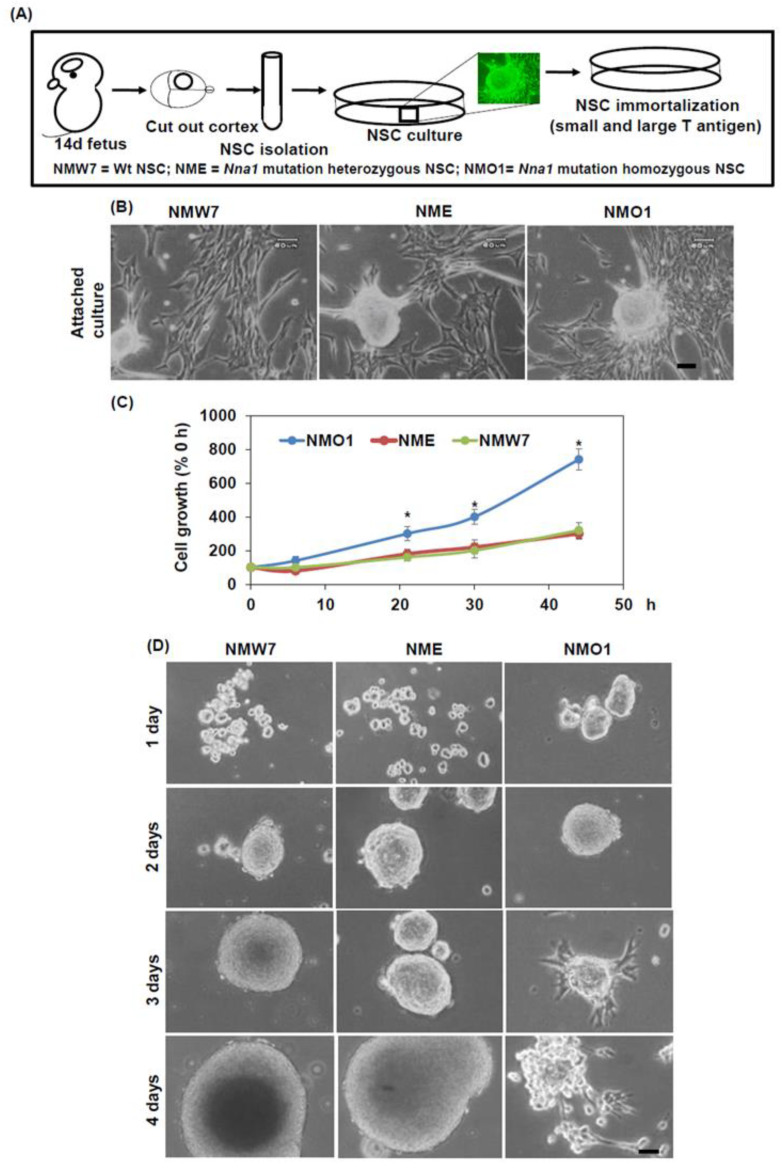
Establishment of the neural stem cell lines: Neural stem cell (NSC) lines were generated from wild-type, *Nna1* mutation heterozygous, and AMS mouse embryos, as described in the Materials and Methods section: (**A**) the schematic design of the procedure to generate the NSC lines, where the neuronal stem cell line NMW7 was generated from wild-type, NME was generated from *Nna1* mutation heterozygous, and NMO1 was generated from AMS mouse embryos. (**B**) The cell morphology of the three cell lines in attached culture condition are shown here. (**C**) NSC cell lines cultured in attached conditions to analyze their growth rate, where viable cell numbers were counted in a time-dependent manner and presented as fold increases compared to an initial number of cells (day 0), and the averages ± SD of 3 independent experiments are shown. (**D**) The results of the neurosphere assay are shown here. NMW7, NME, and NMO1 cells were cultured in neurosphere culture conditions, and neurosphere formation was evaluated in a time-dependent manner using a cell culture microscope. Numerical data are presented as average ± SD of at least 3 independent experiments. Statistical significance is denoted as follows: * *p* < 0.05 vs. NMW7 or NME. Scale bar, 60 μm.

To further investigate the pathology of AMS mice at the molecular and cellular levels, we generated three different NSC lines from embryonic brains of wild-type, *Nna1* mutation heterozygous, and AMS (*Nna1* mutation homozygous) mice, named NMW7, NME, and NMO1, respectively. A schematic representation of the protocol used to generate NSC lines is depicted in Figure 2A. Figure 2B shows representative photomicrographs of NMW7, NME, and NMO1 cells in attached culture condition. The cell size of NMO1 cells was smaller than that of NME or NMW7 cells. Cell growth assays revealed that the growth rate of NMO1 was much higher compared to that of NME or NMW7 (Figure 2C). Neurosphere assays showed that some of the NMO1 neurospheres started to attach to the bottom of the plate from day 3 of the culture and continued to grow as attached cells while the other two cell lines did not show this tendency (Figure 2D).

### 3.3. Characterization NSC Lines

**Figure 3:**
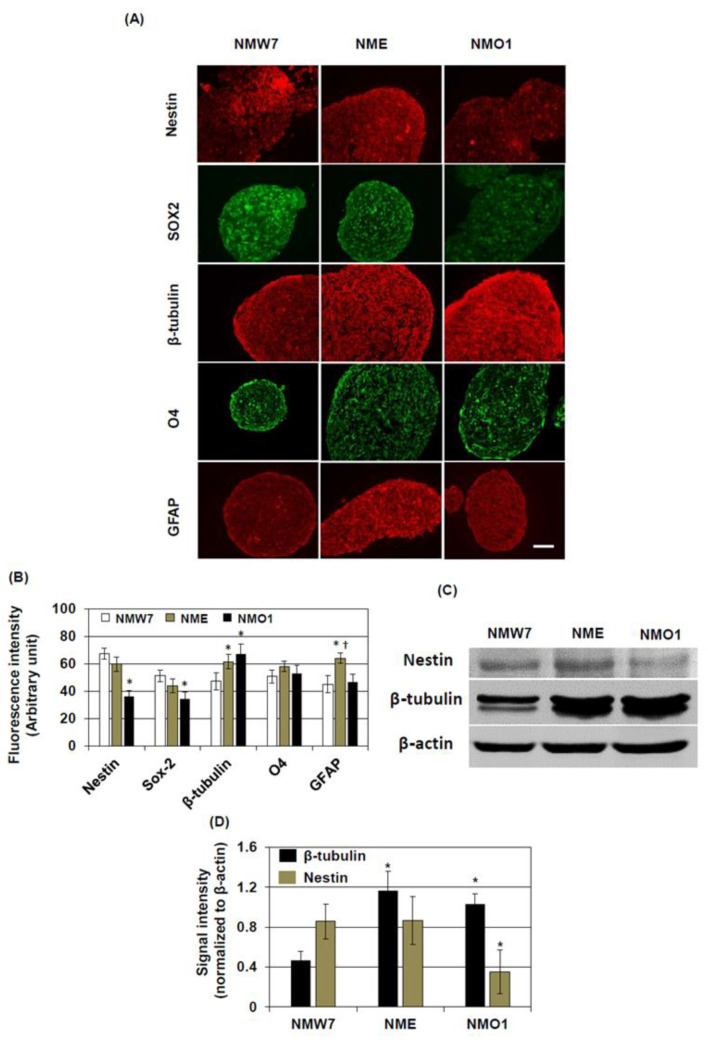
Characterization of neuronal stem cell lines: To characterize NMW7, NME, and NMO1 cells, the protein expression levels of the cell type markers including SOX2, nestin, β-tubulin, O4, and glial fibrillary acidic protein (GFAP) were evaluated under neurosphere culture conditions. (**A**) Representative photomicrographs (×400 magnification) of neurospheres immunostained for the indicated cell type markers are shown here. (**B**) Fluorescence intensities of the cell type markers were quantified as described in the Materials and Methods section, and average fluorescence intensities are shown. (**C**,**D**) The protein expression of β-tubulin and nestin was further evaluated by Western blotting. Representative of Western blotting data are shown in (**C**). β-actin served as the loading control. (**D**) The protein levels were quantified by densitometry, and average densitometric data normalized to β-actin are presented (*n* = 3). Numerical data are presented as average ± SD of at least 3 independent experiments. Statistical significance is denoted as follows: * *p* < 0.05 vs. NMW7; ^†^ *p* < 0.05 vs. NMW7. Scale bar, 50 μm.

Next, we characterized NMW7, NME, and NMO1 by analyzing the protein expression of several NSC markers. The marker expression was analyzed in the neurospheres after 3 days of culture. Since some NMO1 neurospheres were attached at 3 days in culture, we excluded them from this analysis by collecting floating neurospheres only. Immunofluorescence staining of neurospheres demonstrated that, compared to NMW7, the levels of NSC markers including SOX2 and nestin were significantly decreased in NMO1 neurospheres (Figure 3A,B). By contrast, neuronal marker β-tubulin levels were increased in NMO1 and NME neurospheres. In the NME neurospheres, astrocyte marker GFAP levels were increased compared to NMO1 and NMW7 (Figure 3A,B). The pro-oligodendrocyte marker O4 levels were not different among the neurospheres generated from NMW7, NME, or NMO1 (Figure 3A,B). We further checked the levels of nestin and β-tubulin in neurospheres of NMW7, NME, and NMO1 using Western blotting. The results confirmed that nestin levels were significantly decreased in NMO1 neurospheres compared to NMW7 and that β-tubulin levels were increased in NMO1 and NME neurospheres compared to NMW7 (Figure 3C,D).

### 3.4. Posttranslational Modification of β-Tubulin in Neurosphere Culture

**Figure 4:**
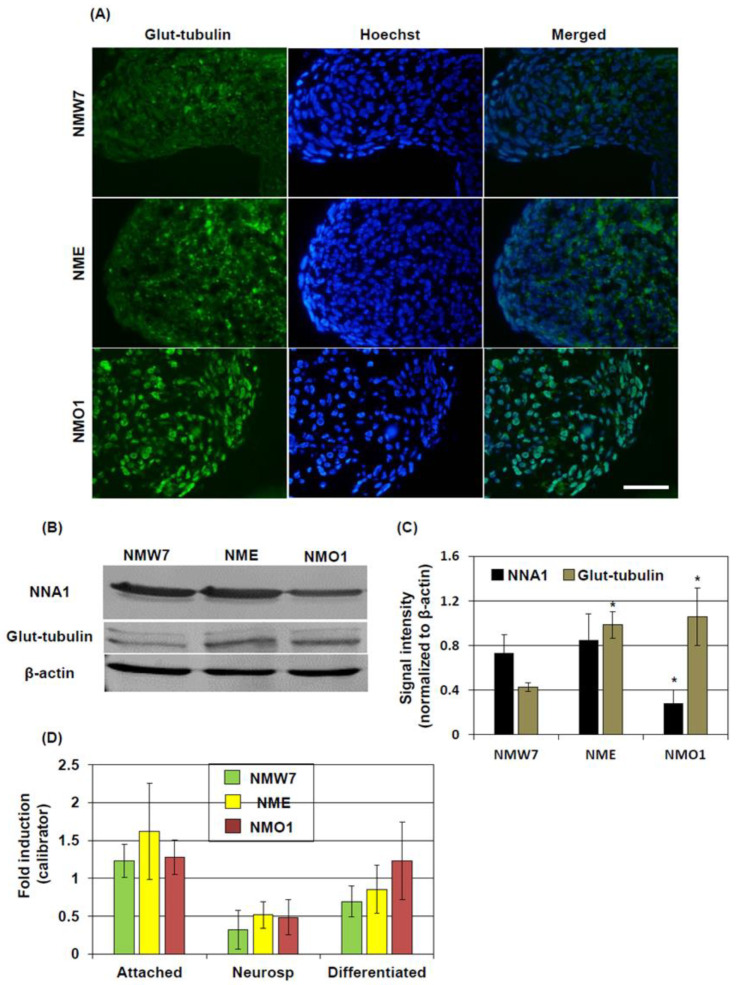
Protein modification and mRNA expression of β-tubulin in NSC culture: Neurospheres were prepared from NMW7, NME, and NMO1 NSCs. (**A**) Glutamylation of tubulin in neurospheres was evaluated by immunofluorescence staining. (**B**,**C**) Glutamylated tubulin and NNA1 levels in neurosphere cultures were further evaluated by Western blotting. (**B**) Representative Western blotting results of glutamylated tubulin and NNA1 are shown here. β-actin was used as a loading control. (**C**) NNA1 and glutamylated tubulin were quantified by densitometry. Averages of the dentisometric data are shown (*n* = 3). (**D**) To evaluate the β-tubulin mRNA levels, total RNA was isolated from NSC attached culture (Attached), neurospheres (Neurosp), and after neuronal differentiation (Differentiated), and real-time PCR was performed as described in the Materials and Methods section. Average ± SD of 3 individual real-time PCR experiments are shown. Statistical significance is denoted as follows: * *p* < 0.05 vs. NMW7. Scale bar = 50 µM.

Previous studies demonstrated that posttranslational modification including glutamylation of β-tubulin can affect microtubule dynamics [25]. Since NNA1 is considered to have deglutamylase activity and the β-tubulin level was increased in AMS-derived NSC lines, we investigated the tubulin glutamylation status in cell culture condition. Immunofluorescence staining results demonstrated that glutamylated tubulin levels were increased in NMO1 and NME neurospheres compared to NMW7, although this difference was not as dramatic as in the case of NME (Figure 4A). Western blotting results confirmed increased levels of glutamylated tubulin in NMO1 and NME neurospheres (Figure 4B,C). The ratio of glutamylated tubulin to β-tubulin was also increased in NMO1 and NME neurospheres (Figure S2). On the other hand, NNA1 protein levels were decreased in NMO1 neurosphere cultures (Figure 4B,C). Since, β-tubulin protein expression was increased in NMO1 and NME neurospheres (see Figure 3), the increase in glutamylated tubulin could be due to the increased transcriptional activity of the *β-tubulin* gene, not altered glutamylation-related enzyme activities. To address this issue, we checked the β-tubulin expression at the mRNA level. The real-time PCR results showed that β-tubulin mRNA levels were not different between NMW7, NME, and NMO1 cell lines in attached culture, neurospheres, or differentiated neurons (Figure 4D).

### 3.5. Neuronal Differentiation Potential of the NSC Lines

**Figure 5:**
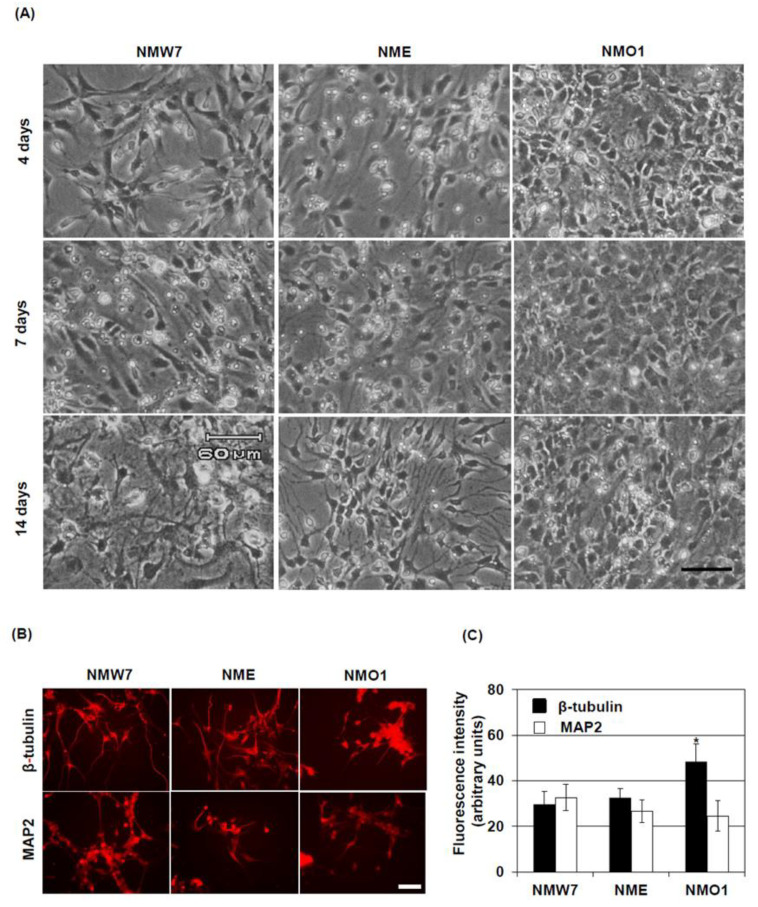
Differentiation of the generated neural stem cell lines: Neuronal differentiation was induced in NMW7, NME, and NMO1 cells using differentiation culture medium and poly-L-lysine coated plates, as described in the Materials and Methods section. Time-dependent morphological changes of the cell lines during differentiation in culture were evaluated using a cell culture microscope. (**A**) Representative photomicrographs of NMW7, NME, and NMO1 cells under neuronal differentiation conditions at 4, 7, and 14 days in culture are shown here. Scale bar = 60 µm. (**B**,**C**) Protein expressions of β-tubulin and microtubule-associated protein 2 (MAP2) after differentiation were evaluated using immunofluorescence staining. (**B**) Representative photomicrographs of β-tubulin and MAP2 immunofluorescence staining of NMW7, NME, and NMO1 cells after neuronal differentiation are shown here. Scale bar = 50 µm. (**C**) Fluorescence intensities of β-tubulin and MAP2 were quantified as described in the Materials and Methods section, and average fluorescence intensities are shown. Numerical data are presented as averages ± SD of 3 independent experiments. Statistical significance is denoted as follows: * *p* < 0.05 vs. NMW7.

Next, we investigated the differentiation potential of the NSC lines to mature neurons. After 4 days in culture under neuron differentiation conditions, NMW7 and NME started to extend processes from the cell body. After 7 days in culture, cell division was decreased, and well-defined processes originated from the cell body. At 14 days, the cell growth of NMW7 and NME had almost stopped, and extensive processes emerged from the cell body (Figure 5A). In the case of NMO1, cell division was slightly decreased at 7 days in culture and the processes were not as well-defined as in NMW7 or NME cultures. At 14 days in culture, cell numbers were still increasing in NMO1 culture and the processes were found to be smaller in size than those of NMW7 or NME cells (Figure 5A). Immunostaining of β-tubulin showed that, after differentiation, the cells have long processes in both NME and NMW7 culture, whereas cell processes were short in NMO1 cultures (Figure 5B). Moreover, the intensity of β-tubulin staining was high in NMO1 cultures (Figure 5C). Western blotting results also confirmed significant increased β-tubulin levels in NMO1 neuron differentiated culture (Figure S3). In comparison, MAP2 staining pattern and fluorescence intensities were found to be similar in NMO1, NME, and NMW7 culture (Figure 5B,C).

### 3.6. β-Tubulin Protein Expression and Posttranslational Modification in the Cerebellum of AMS Mice

**Figure 6:**
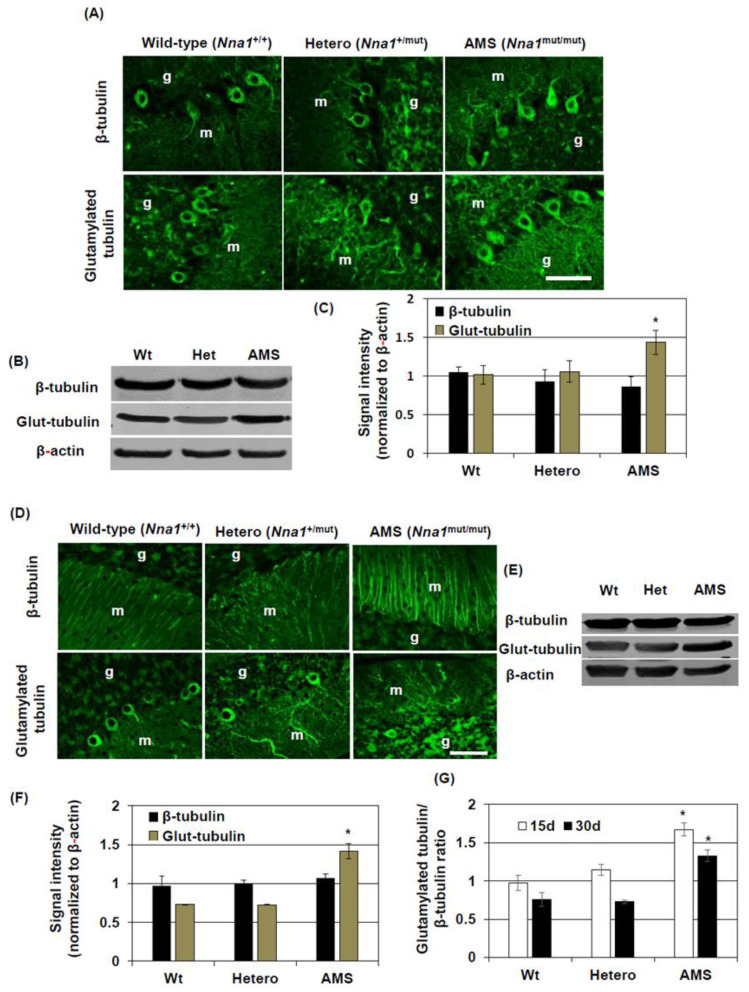
β-tubulin protein expression and modification in the cerebellum of AMS mice: Protein expression and glutamylation of β-tubulin in the cerebellum were evaluated using immunofluorescence staining and Western blotting. Representative photomicrographs of β-tubulin and glutamylated tubulin (Glut-tubulin) immunofluorescence staining of the cerebellum of wild-type, *Nna1* mutation heterozygous (Hetero), and AMS mice at 15 (**A**) and 30 (**D**) days of age are shown. m = molecular layer, g = granular layer. β-tubulin and glutamylated tubulin levels were further evaluated by Western blotting. Representative Western blotting results of the cerebellum of wild-type (Wt), *Nna1* mutation heterozygous (Het), and AMS mice at 15 (**B**) and 30 (**E**) days of age are displayed. β-actin was used as a loading control. The levels of β-tubulin and glutamylated tubulin were quantified by densitometry, and the quantified data normalized to β-actin of mice at an age of 15 (**C**) and 30 (**F**) days are shown. The ratio of glutamylated tubulin to β-tubulin are shown in (**G**). Numerical data are presented as averages ± SD of 5 mice in each group. Statistical significance is denoted as follows: * *p* < 0.05 vs. wild-type. Scale bar = 50 µM.

Since the β-tubulin protein level was increased in NMO1 cells, we investigated protein levels and posttranslational modification of β-tubulin in the cerebellum of AMS mice. Immunofluorescence staining results revealed that β-tubulin was mainly expressed in the cell body and proximal processes of cerebellar PCs of AMS, *Nna1* mutation heterozygous, and wild-type mice cerebellum at 15 days of age (Figure 6A and Figure S4). Some of the cells in the granular layer were also positive for β-tubulin. At the age of 30 days, long processes in the molecular layer were positive for β-tubulin along with some cell bodies in the granular layer (Figure 6A). Western blotting results showed that the β-tubulin protein level was unchanged in the cerebellum of AMS mice at both 15 and 30 days (Figure 6B,C,E,F). However, long processes in the molecular layer of AMS mice stained strongly for β-tubulin at 30 days (Figure 6D and Figure S5).

Next, we checked the glutamylation status of tubulin in the cerebellum of AMS mice. Immunofluorescence staining results demonstrated that glutamylated tubulin was mainly present in the cell bodies and proximal processes of PCs in both 15- and 30-day-old wild-type mice (Figure 6A,D and Figures S4 and S5). In AMS mice, the staining pattern was similar to that of wild-type mice at 15 days (Figure 6A and Figure S4). Since almost complete PC degeneration occurred in AMS mice at 30 days, very few numbers of cell bodies were found to be positive for glutamylated tubulin. However, some processes in the molecular layer still stained positive for glutamylated tubulin (Figure 6D and Figure S5). Besides, the cell bodies of granular layer were strongly positive for glutamylated tubulin in AMS mice at 30 days of age. Western blotting results showed that, at both 15 and 30 days, the levels of glutamylated tubulin were significantly increased in AMS mice cerebellum (Figure 6B–F).

### 3.7. MAP2 Protein Expression in the Cerebellum of AMS Mice

**Figure 7:**
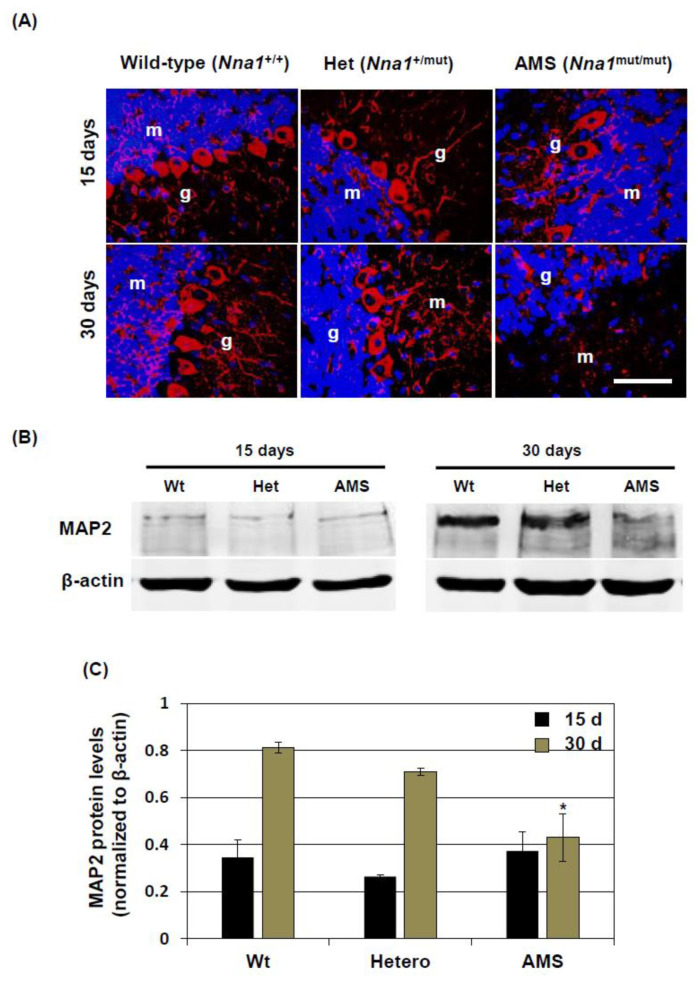
MAP2 protein expression in the cerebellum of AMS mice: The protein expression of MAP2 was evaluated by immunofluorescence staining and Western blotting. (**A**) Representative photomicrographs of MAP2 immunofluorescence staining of wild-type, *Nna1* mutation heterozygous (Het), and AMS mice cerebellum at 15 (upper row) and 30 (lower row) days of age are shown. Nuclei were stained with Hoechst. m = molecular layer, g = granular layer. (**B**,**C**) MAP2 expression was further evaluated by Western blotting. (**B**) Representative MAP2 Western blotting results of cerebellar samples from wild-type (Wt), *Nna1* mutation heterozygous (Het), and AMS mice at 30 days of age are shown here. β-actin served as a loading control. (**C**) MAP2 protein levels at 15 and 30 days of age were quantified by densitometry, and the data normalized to β-actin are shown here. Numerical data are presented as averages ± SD of 5 mice in each group. Statistical significance is denoted as follows: * *p* < 0.05 vs. wild type. Scale bar = 50 µM.

To further investigate microtubules, we analyzed MAP2 protein expression in the cerebellum of AMS mice. Immunofluorescence staining results revealed that the cell bodies of PCs were positive for MAP2 protein in wild-type mice at both15 and 30 days (Figure 7A and Figure S6). Moreover, some cell bodies and processes in the molecular layer were MAP2 positive, along with some areas in the granular layer. The granular layer was identified by Hoechst-stained densely packed nuclei. A similar staining pattern was observed in the cerebellum of *Nna1* mutation heterozygous mice. In AMS mice at 15 days, a few PCs were found to be positive along with some areas in the molecular and granular layer. At 30 days, MAP2-positive cells in the molecular layer were rare (Figure 7A and Figure S6). Western blotting results demonstrated that, compared to 15 days, MAP2 protein levels were increased in wild-type cerebellum at 30 days of age. Interestingly, MAP2 protein levels at 30 days were significantly decreased in the cerebellum of AMS mice compared to those of wild-type or *Nna1* mutation heterozygous mice (Figure 7B,C).

## 4. Discussion

In our previous study, we found that dying PCs in AMS mice present some morphological features of apoptosis at 3 weeks of age [3]. Since NNA1 has a deglytamylase activity and β-tubulin can be glutamylated posttranslationally [12], we considered β-tubulin glutamylation as a target of further investigation to elucidate the mechanism of cell death in this mouse. In this study, we demonstrated that the glutamylated tubulin level was increased in the cerebellum of AMS mice. Cytoskeletal proteins like actins or tubulins can undergo several types of posttranslational modifications including acetylation, argenylation, oxidation, and phosphorylation [17,18,26,27]. Such posttranslational modifications are important because precise modifications are essential for proper functioning of the cytoskeleton. Among the posttranslational modifications of cytoskeletal proteins, glutamylation was only observed in tubulins. Other cytoskeletal proteins like β-actin did not undergo deglutamylation, suggesting that, among cytoskeletal proteins, tubulin could be the only target of NNA1 deglutamylase activity. Since a missense mutation was described in the *Nna1* gene [4], increased glutamylated tubulin levels could result from the defective function of this deglutamylase in AMS mice. A similar finding was reported in PCD mice, where its loss-of-function due to mutation caused increased tubulin glutamylation [14]. The increased glutamylation alters microtubule dynamics and functions, and this is deleterious for PCs and retinal photoreceptors [14,28]. As a mechanism, it was suggested that hyperglutamylation could lead to mitochondrial fragmentation resulting in PC degeneration [14]. Moreover, in the retina, hyperglutamylation causes decreased glycylation of tubulin, and the subsequent imbalance of the glutamylation-glycylation equilibrium leads to retinal degeneration [28]. In the present study, we demonstrated that increased glutamylation may lead to defective functions of neural stem cells in terms of their growth and differentiation. Such functional defects might lead to impaired maturation and ultimately PC degeneration. Nevertheless, it will be interesting to know the detailed mechanisms by which tubulin glutamylation affects cell death processes like apoptosis.

In AMS mice, homozygous mutation of the *Nna1* gene was found, causing an amino acid change in the protein sequence at position 808. Previous studies showed that amino acid changes in this region of the protein (686 Y to D, 843 T to M, and 910 R to W) crucially affect the deglutamylation activity [22]. Moreover, in AMS mice, cerebellar total NNA1 protein levels were found to be decreased at the age of 15 days and older. At the age of 30 days, AMS mice did not present NNA1 stained PC due to their degeneration, although the staining patterns did not differ much among the strains at 15 days of age. The reason for reduction is not fully elucidated, but a probable cause could be the decreased translation or stability of the protein induced by the mutation [4]. Nevertheless, this reduced expression might also contribute to decreased deglutamylation activity. Since, *Nna1* is highly expressed in developing neurons [29], it is conceivable that it has a role in maturation of the neurons. Hence, due to decreased protein level and reduced deglutamylation activity caused by the mutation, the maturation of neurons could be affected in AMS mice.

As demonstrated in our previous report, the overall size of the cerebellum is decreased in 30-day-old mice, although at 15 days, the cerebellar size of AMS mice is similar to that of wild-type mice [1]. During the first 2 weeks, profound morphological and functional changes occur in rodent PCs [30]. PC degeneration starts in AMS mice just after these morphological changes. Migration, maturation, and synaptic contacts of cerebellar granule neurons occur during the first 3 weeks of a mouse pup’s life [31]. Since the volume of granular layer is decreased just after this time point and the overall size of the cerebellum is decreased, the maturation process of cerebellar neurons could also be altered in AMS mice. Hence, the *Nna1* mutation may cause disturbances in the function of immature neurons or neural stem cells. Consistent with that hypothesis, our in vitro cell culture system demonstrated that the properties of an NSC line derived from AMS (NMO1) are different from those derived from wild-type or *Nna1* mutation heterozygous mice, such as decreased nestin and SOX2 levels in the neurosphere. Nestin and SOX2 are widely considered as neural stem cell markers. Several reports have demonstrated their essential role during self-renewal process of NSCs [32,33], and their expressions are decreased during differentiation into neurons or glia. Since nestin and SOX2 are considered to be involved in maintaining stem cell properties, decreased nestin and SOX2 in NMO1 neurospheres might imply that the stemness of NSCs in this mouse strain was decreased due to the *Nna1* mutation. On the other hand, the neuronal marker β-tubulin level was increased in NMO1 neurospheres, suggesting that the NSC might spontaneously differentiate to neurons in neurosphere culture condition. Attachment of these neurosphere to the bottom of the culture plates also suggests the same. However, neuron differentiation experiments do not support the idea that NMO1 has an improved differentiation ability. For maturation of neurons, proper and balanced posttranslational modifications of microtubule proteins are essential. Since glutamylation is increased in NSC generated from AMS, such a balanced could be disturbed. A previous study showed that tubulin hyperglutamylation increases the stability of microtubules in the cell body and decreases it in the axoneme [34]. Since β-tubulin expression was not changed at the transcriptional level among the NSC lines, stabilization of microtubules in the cell body could be the reason for the increased β-tubulin level in AMS NSCs. In differentiated neurons, the β-tubulin level was found to be increased in the cell body. However, the processes seem smaller in length in neurons differentiated from the AMS-derived NSC line. Interestingly, The NSC line derived from *Nna1* mutation heterozygous mice also showed increased β-tubulin levels in neurosphere cultures, but in cultured mature neurons, the length of processes and β-tubulin levels seemed to be similar to those in neurons derived from wild-type NSCs. Since half of the protein should function normally in this cell line, these findings could highlight the importance of functional NNA1 levels in microtubule physiology during neuronal development.

In contrast to cell culture, the total β-tubulin level did not change in the cerebellum of AMS mice as shown by Western blotting. However, immunostaining results showed that β-tubulin expression was increased in the processes of the molecular layer. This result could indicate a differential β-tubulin regulation in the cerebellum in vivo compared to culture conditions. However, a strong β-tubulin immunostaining signal in the molecular layer of AMS mice may also suggests that, although overall β-tubulin level was not changed, it indeed increased in some types of cells where NNA1 expression is considered high. NNA1 is expressed highly in cerebellar PCs [8]. Hence, a decreased function of NNA1, evidenced by increased tubulin glutamylation, might alter β-tubulin stability and microtubule regulation in the PCs. In other cell-types, due to decreased NNA1 expression, this regulation might not come into play at this stage, resulting in a similar overall β-tubulin turnover in the cerebellum of AMS mice. On the other hand, MAP2 expression is increased in mature neurons. In AMS mice, the MAP2 protein level was decreased, further confirming the deregulation of microtubule assembly in this mouse strain. This deregulation could induce PC degeneration. Since NNA1 is also expressed in granule cells, these cells as well are vulnerable to the deregulated microtubule assembly due to loss-of-function of NNA1.

In this study, we found that the NSC line generated from AMS mice grows faster than those of wild-type or *Nna1* mutation heterozygous mice. In the neuronal cells, microtubules can be found not only in the cell body and processes but also in centrioles and mitotic spindle. In a previous report, it was shown that, during the mitotic process, glutamylation of β-tubulin is increased in centrioles and mitotic spindles [35]. Due to defective NNA1 functions, glutamylated β-tubulin levels might be increased in the cytoplasm and is therefore responsible for the increased growth of the AMS-derived NSC line. This accelerated growth might also disrupt the differentiation process, as seen in our neuronal differentiation culture.

## 5. Conclusions

In conclusion, our result demonstrated that *Nna1* mutation in AMS mice might alter NNA1 function, leading to increased β-tubulin glutamylation and stability as well as decreased MAP2 expression. The alteration of microtubule-related proteins could affect microtubule assembly and function. Due to high NNA1 expression in PCs, this deregulation of microtubules may profoundly affect the maturation of these cells and induces their degeneration in AMS mice.

## Data Availability

Any additional data will be available upon request to the corresponding author.

## Supplementary Materials

The following are available online at https://www.mdpi.com/2073-4409/10/1/155/s1, Figure S1: Calibration curve for Western blotting; Figure S2: Ratio of glutamylated tubulin and β-tubulin in neurosphere culture; Figure S3: β-tubulin protein expression in NSC differentiated to neurons in culture; Figure S4: β-tubulin and glutamylated tubulin immunostaining (15 days old mice); Figure S5: β-tubulin and glutamylated tubulin immunostaining (30 days old mice); Figure S6: MAP2 protein expression in the mice cerebellum evaluated by immunostaining.
