# Supplementary material for: Alteration of Neural Stem Cell Functions in Ataxia and Male Sterility Mice: A Possible Role of β-Tubulin Glutamylation in Neurodegeneration
Source: Cells. 2021 Jan 14;10(1):155. doi: 10.3390/cells10010155 (PMC7830091; doi:10.3390/cells10010155)
Supplement: Supplementary file 1 [file cells-10-00155-s001.pdf]

Alteration of Neural Stem Cell Functions in Ataxia and Male Sterility Mice: A Possible Role of β-Tubulin Glutamylation in Neurodegeneration

Abdullah Md. Sheikh [1,*], Shozo Yano [1], Shatera Tabassum [1], Koji Omura [2], Asuka Araki [2], Shingo Mitaki [3], Yoshie Ito [3], Shuai Huang [4] and Atsushi Nagai [3]

[1] Department of Laboratory Medicine, Shimane University Faculty of Medicine, 89-1 Enya Cho, Izumo 693-8501, Japan; syano@med.shimane-u.ac.jp (S.Y.); tabassum@med.shimane-u.ac.jp (S.T.)
[2] Department of Organ Pathology, Shimane University Faculty of Medicine, 89-1 Enya Cho, Izumo 693-8501, Japan; kou00@med.shimane-u.ac.jp (K.O.); asuka@med.shimane-u.ac.jp (A.A.)
[3] Department of Neurology, Shimane University Faculty of Medicine, 89-1 Enya Cho, Izumo 693-8501, Japan; shingomi@med.shimane-u.ac.jp (S.M.); yo.ito@med.shimane-u.ac.jp (Y.I.); anagai@med.shimane-u.ac.jp (A.N.)
[4] Department of Orthopedic Surgery, The Second Affiliated Hospital of Guangzhou Medical University, 250 Changgangdong Road, Guangzhou 510260, China; huang-shuai@hotmail.com
* Correspondence: abdullah@med.shimane-u.ac.jp; Tel.: +81-0853-20-2306

Supplemental Methods and Results

Calibration curve for Western blotting

Since we used mainly Western blotting for quantification of protein-of-interest in our study, we validated our quantification method by preparing a calibration curve for cerebellar proteins isolated from both wild-type and AMS mice. In the wells of 4-20% SDS polyacrylamide gel, 40, 20, 10, 5, 2.5 and 0 μg of total protein of wild-type and AMS mice were applied and Western blotting was performed for β-tubulin and β-actin. The results showed that β-tubulin and β-actin produced a linear curve when wild-type cerebellar protein was used (r2 value: 0.9967 and 0.9868, respectively) (supplemental figure 1A, 1B, 1C and 1E). Similarly, β-tubulin and β-actin produced a linear curve when AMS cerebellar protein was used (r2 value: 0.9889 and 0.9948, respectively) (supplemental figure 1A, 1B, 1D and 1F). Analyzing the ratio of β-tubulin and β-actin revealed that the ratio was stable up to 5 μg of protein, below that level the ratio started to fluctuate (supplemental figure 1G). Since, we used 20-60 μg protein, this result suggests that precise protein quantification by Western blotting is achievable.

Ratio of glutamylated tubulin to β-tubulin is increased in the neurosphere culture condition

We have analyzed the levels of β-tubulin and glutamylated tubulin in neurosphere culture condition by Western blotting. β-tubulin levels in the neurospheres of NMW7 NME and NMO1 are shown in figure 3C and 3D, and that of glutamylated tubulin levels in figure 4B and 4C. In the supplemental figure 2, we showed the ratio of glutamylated tubulin and β-tubulin. The results showed that the ratio of glutamylated tubulin/ β-tubulin was significantly increased NME and NMO1 compared to NMW7.

Protein expression of β-tubulin in NSC culture after neuron differentiation

Here we have investigated the protein expression β-tubulin after differentiation of NSC to mature neurons in culture. The Western blotting results showed that β-tubulin levels in neuron differentiated NMW7 and NME are similar. However, β-tubulin expression in neuron differentiated NMO1 was significantly increased, similar to the results shown using immunocytochemistry. The results are shown in supplemental figure 3.

β-tubulin protein expression and posttranslational modification in the cerebellum of AMS mice

Here we showed the immunostaining data of β-tubulin and glutamylated tubulin expressed in the cerebellum of wild-type, *Nna1* mutation heterozygous and AMS mice with a larger field of area, as a supplement to figure 6. In the figure 4, β-tubulin and glutamylated tubulin immunostaining data of wild-type, Nna1 mutation heterozygous and AMS mice at 15 days of age are shown. In the figure 5, β-tubulin and glutamylated tubulin immunostaining data of wild-type, Nna1 mutation heterozygous and AMS mice at 30 days of age are shown.

MAP2 protein expression in the cerebellum of AMS mice

In the supplemental figure 6, we showed the immunostaining data of MAP2 expressed in the cerebellum of wild-type, *Nna1* mutation heterozygous and AMS mice with a larger field of area, as a supplement to figure 7. The results showed that in the cerebellum of wild-type mice at 15 days of age, MAP2 levels in the molecular layer were much lower than that at 30 days of age. *Nna1* mutation heterozygous mice also showed similar pattern of staining. In AMS mice, MAP2 expression in the molecular layer at 15 days of age was similar line wild-type or *Nna1* mutation heterozygous mice. However, at 30 days of age, MAP2 levels were decreased in the molecular layer of AMS mice compared to wild-type or *Nna1* mutation heterozygous mice.

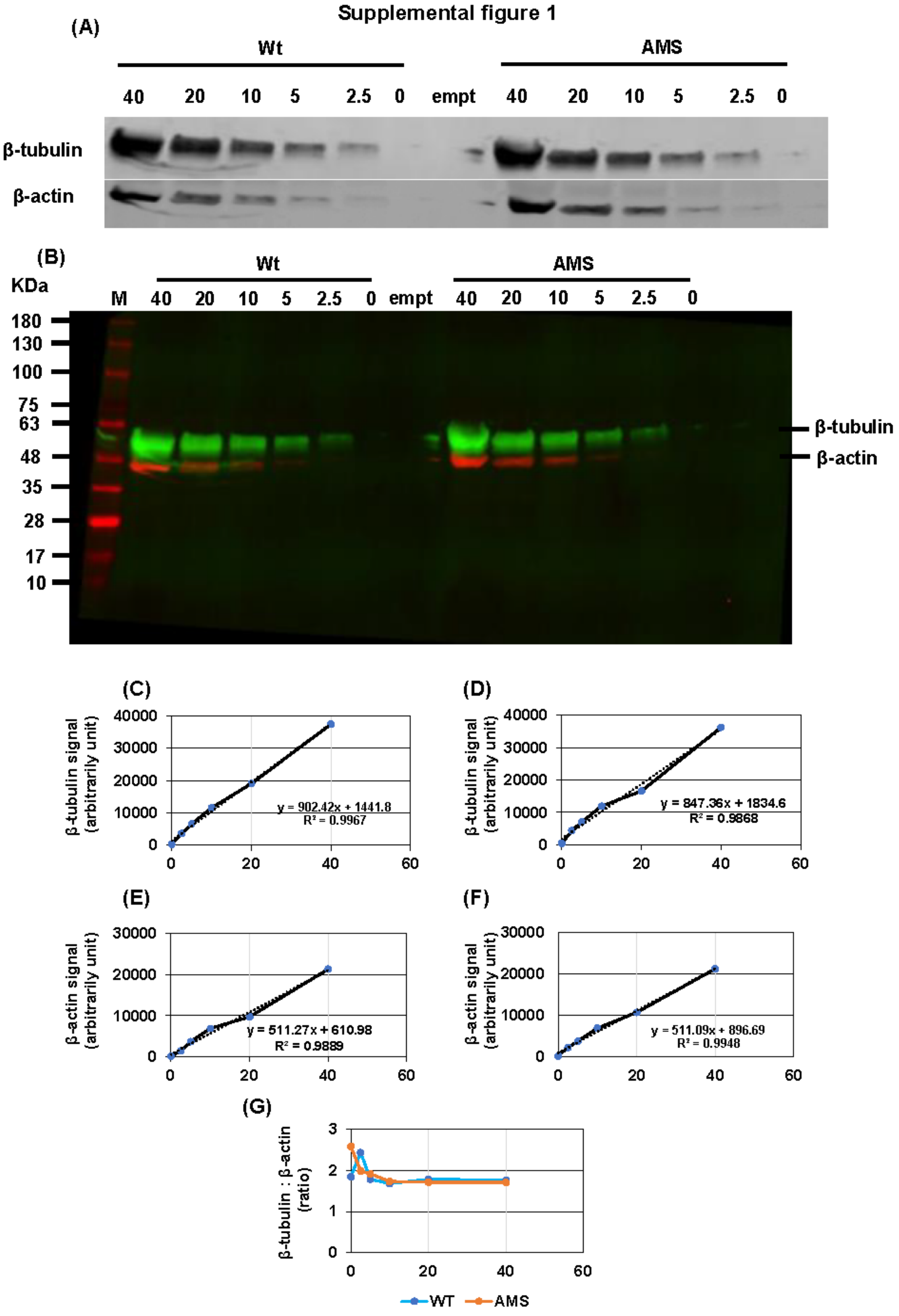

Supplemental figure 1: Calibration curve for Western blotting: To prepare calibration curve for β-tubulin and β-actin Western blotting was done using 40, 20, 10, 5, 2.5 and 0 μg of total protein isolated from the cerebellum of 1 wild-type and 1 AMS mouse cerebellum at 30 days of age. After SDS PAGE, the protein was transferred to a PVDF membrane and simultaneously immunoblotted with anti-β-tubulin and anti-β-actin antibodies. A representative immunoblot picture of 3 independent

experiments are shown in (A), where the upper panel is the immunoblot of β-tubulin and lower panel is the immunoblot of β-actin. Figure (B) is the combined immunoblot of β-tubulin and β-actin from where the blots of (A) were generated. Green color indicates the immunoblot of β-tubulin, and red color is that of β-actin. M indicates the lane for protein molecular weight size marker. Immunoreactive bands were quantified by densitometry using ImageJ. Using densitometric data, calibration curves for β-tubulin (C and D) and β-actin (E and F) were made, where (C) and (E) are the calibration curves of wild-type mouse, and (D) and (F) are the calibration curves of AMS mouse sample. The ratio of β-tubulin and β-actin is shown in (G).

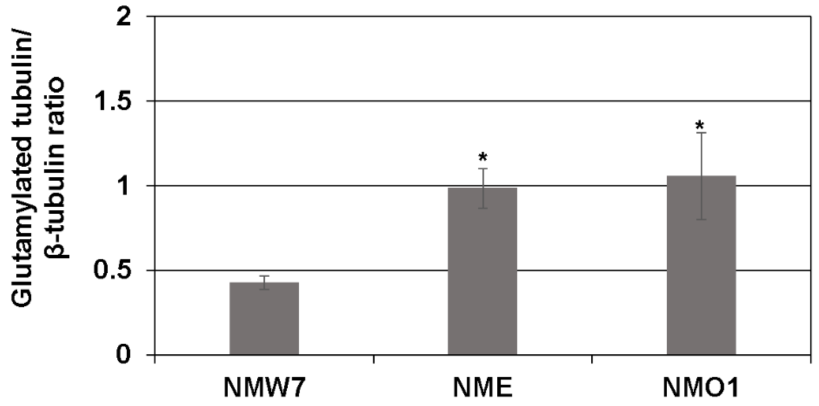

Supplemental figure 2. Ratio of glutamylated tubulin and β-tubulin. Glutamylated tubulin and β-tubulin in NMW7, NME and NMO1 neurospheres were evaluated by Western blotting. The levels of the proteins were quantified by densitometry. The ratio of glutamylated tubulin and β-tubulin was calculated, and average of the ratio of 3 independent experiments are shown here. *$p < 0.05$ vs NMW7.

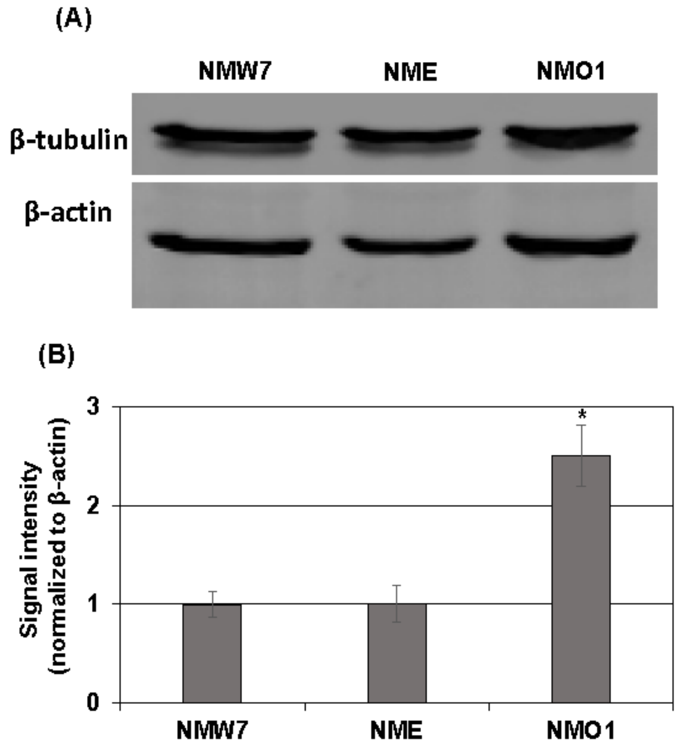

Supplemental figure 3. β-tubulin protein expression in NSC differentiated to neurons in culture. Neurospheres generated from NMW7, NME and NMO1 NSC were plated in Poly-L-lysine-coated cell cultured dishes, and cultured in neuron differentiation medium for 14 days. Total protein was isolated and Western blotting was done to evaluate the levels of β-tubulin. As a loading control, β-actin immunoblotting of the same membrane was done. Representative immunoblot data are shown

in (A). The blot was further analyzed by densitometry, and the data of 3 independent experiments were averaged and shown in (B). *p< 0.05 vs NMW7.

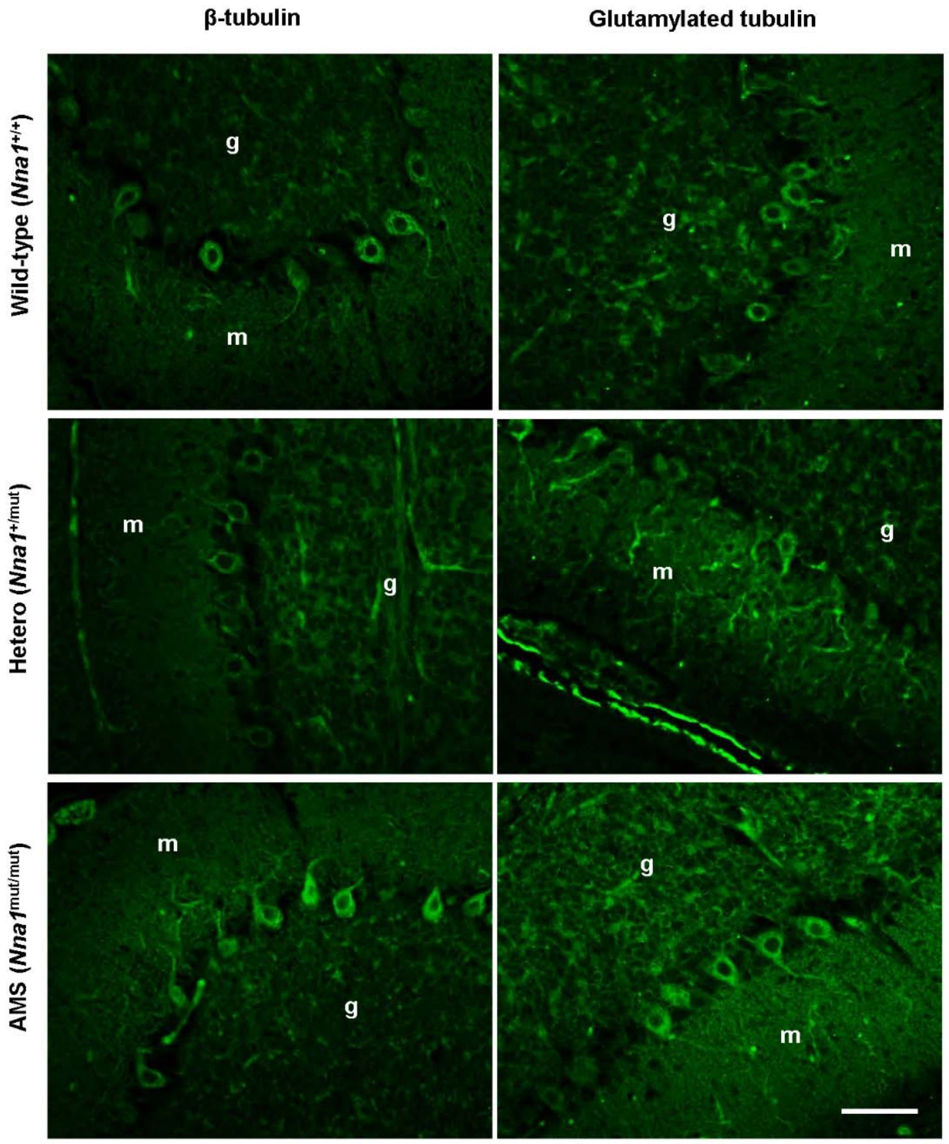

Supplemental figure 4. β-tubulin and glutamylated tubulin immunostaining. β-tubulin and glutamylated tubulin levels in wild-type Nna1 mutation heterozygous and AMS mouse cerebellum were evaluated using immunofluorescence staining. Representative photomicrographs of β-tubulin and glutamylated tubulin (Glut-tubulin) immunofluorescence staining of the cerebellum of wild-type, Nna1 mutation heterozygous (Hetero) and AMS mice at 15 days of age are shown. m= molecular layer, g= granular layer. Scale bar= 50 μM.

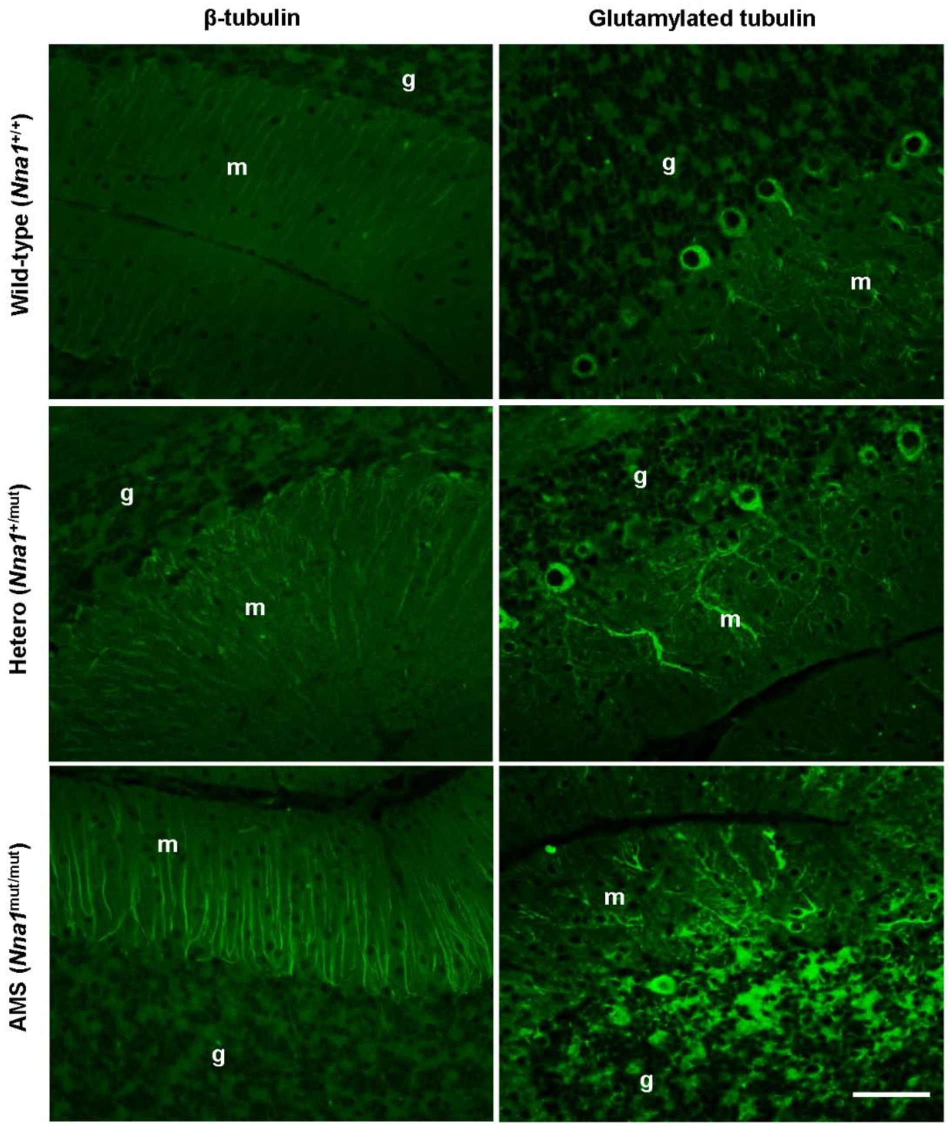

Supplemental figure 5. β-tubulin and glutamylated tubulin immunostaining. β-tubulin and glutamylated tubulin levels in wild-type Nna1 mutation heterozygous and AMS mouse cerebellum were evaluated using immunofluorescence staining. Representative photomicrographs of β-tubulin and glutamylated tubulin (Glut-tubulin) immunofluorescence staining of the cerebellum of wild-type, Nna1 mutation heterozygous (Hetero) and AMS mice at 30 days of age are shown. m= molecular layer, g= granular layer. Scale bar= 50 μM.

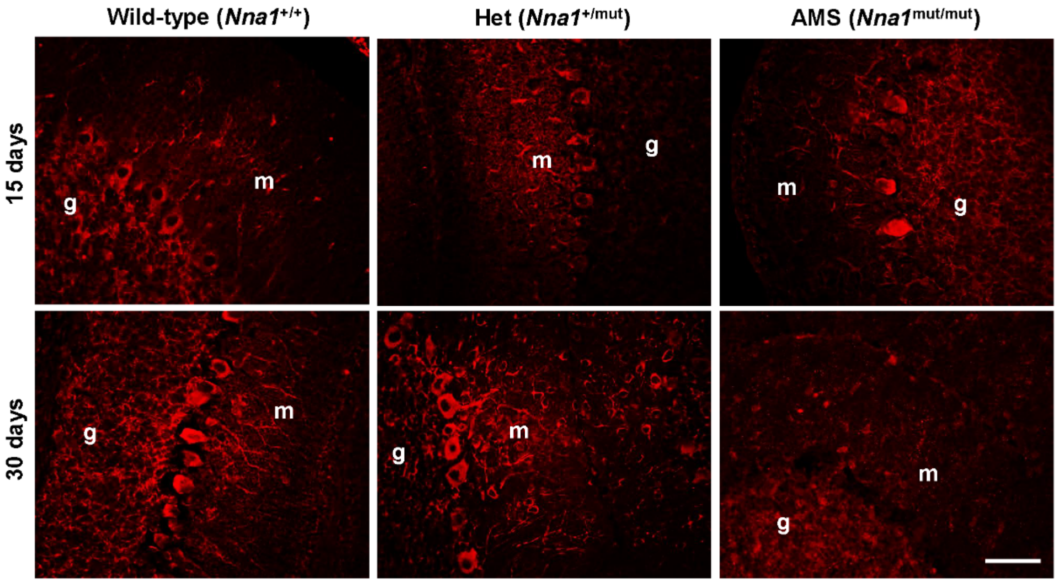

Supplemental figure 6. MAP2 protein expression in the cerebellum evaluated by immunostaining. The protein expression of MAP2 was evaluated by immunofluorescence staining. Representative photomicrographs of MAP2 immunofluorescence staining of wild-type, Nna1 mutation heterozygous (Het) and AMS mice cerebellum at 15 (upper row) and 30 (lower row) days of age are shown. m= molecular layer, g= granular layer. Scale bar= 50 μM.
